# Correlations between Psychopathological Symptoms and Self-stigmatization of Patients with Bipolar Affective Disorder at the Initial Stage of the Disease

**DOI:** 10.1192/j.eurpsy.2025.2149

**Published:** 2025-08-26

**Authors:** V. Mitikhin, G. Tiumenkova, D. Oshevsky

**Affiliations:** 1 Mental Health Research Centre, Moscow, Russian Federation

## Abstract

**Introduction:**

It was demonstrated in the number of studies that patients with bipolar affective disorder (BD) at the initial stage of the disease are characterized by the high level of self-stigmatization (Latalova K., Kamaradova D., Prasko J., 2014; Solokhina T.A., Oshevsky D.S., Barkhatova A.N., et al., 2023). However, relationship of patients’ self-stigmatization with their psychopathological symptoms wasn’t analyzed, what determined the theoretical and practical significance of our study.

**Objectives:**

To reveal correlations between psychopathological symptoms and self-stigmatization of patients with BD at the initial stage of the disease and to work out the integrated approach to their psychosocial treatment.

**Methods:**

Questionnaire for assessing the phenomenon of self-stigmatization of mentally ill people (Mikhailova et al., 2005), SCL-90-R were used. A group of 17 patients (12 women and 5 men) with diagnosis of bipolar affective disorder (BD, F31.xxx according to ICD-10) was examined. The average age of the patients was 25.52±4.55 years. The duration of the disorder varied within 0.5 -3 years.

**Results:**

Patients with BD demonstrated high overall level of self-stigmatization (1.22±0.73 points). This parameter was significantly higher than average values. As a result of correlation analysis, multiple dependable (p<0.01) moderate relationships between SCL-90-R indicators and parameters of several scales of the self-stigmatization questionnaire were established. So, perception of changes associated with the disease as irreversible, depriving opportunities in various spheres of life (scales «Overestimation of self-realization», «Overestimation of internal activity») leaded to somatization of patients and the formation of hypochondriac experiences (SOM, r=0.58 and r=0.54, respectively). In turn, this reduces self-esteem and causes an increase in the overall level of experienced distress (GSI, r=0.61 and r=0.53, respectively). Self-doubt, the expectation of a negative attitude towards oneself (the scale «De-identification from others in the social sphere») leads to increased anxiety (ANX, r=0.64), hostility (HOS, r=0.53), vulnerability in communication and restriction of social contacts (INT, r=0.51).

**Conclusions:**

The obtained results permitted to work out proposals on psychosocial treatment of patients with BD at the initial stage of the disease. It is necessary to carry out psychoeducation programs as well as trainings aimed at forming a positive self-perception, activating personal resources, increasing communicative competence and maintaining social interaction.

**Disclosure of Interest:**

None Declared

